# INVESTIGATION INTO P2Y RECEPTOR FUNCTION IN PLATELETS FROM PATIENTS WITH SEPSIS

**DOI:** 10.1097/SHK.0000000000002158

**Published:** 2023-06-30

**Authors:** Kate L. Arkless, Matthew Fish, Aislinn Jennings, Clive P. Page, Manu Shankar-Hari, Simon C. Pitchford

**Affiliations:** ∗ Sackler Institute of Pulmonary Pharmacology, Institute of Pharmaceutical Science, King’s College London, London, United Kingdom; † School of Immunology and Microbial Sciences, King’s College London, London, United Kingdom; ‡ Centre for Inflammation Research, The University of Edinburgh, Edinburgh, United Kingdom

**Keywords:** Platelets, P2Y_1_, P2Y_12_, chemotaxis, aggregation, sepsis, pneumonia

## Abstract

Key underlying pathological mechanisms contributing to sepsis are hemostatic dysfunction and overwhelming inflammation. Platelet aggregation is required for hemostasis, and platelets are also separately involved in inflammatory responses that require different functional attributes. Nevertheless, P2Y receptor activation of platelets is required for this dichotomy of function. The aim of this study was to elucidate whether P2YR-dependent hemostatic and inflammatory functions were altered in platelets isolated from sepsis patients, compared with patients with mild sterile inflammation. Platelets from patients undergoing elective cardiac surgery (20 patients, 3 female) or experiencing sepsis after community-acquired pneumonia (10 patients, 4 female) were obtained through the IMMunE dysfunction and Recovery from SEpsis-related critical illness in adults (IMMERSE) Observational Clinical Trial. *In vitro* aggregation and chemotaxis assays were performed with platelets after stimulation with ADP and compared with platelets isolated from healthy control subjects (7 donors, 5 female). Cardiac surgery and sepsis both induced a robust inflammatory response with increases in circulating neutrophil counts with a trend toward decreased circulating platelet counts being observed. The ability of platelets to aggregate in response to *ex vivo* ADP stimulation was preserved in all groups. However, platelets isolated from patients with sepsis lost the ability to undergo chemotaxis toward *N*-formylmethionyl-leucyl-phenylalanine, and this suppression was evident at admission through to and including discharge from hospital. Our results suggest that P2Y_1_-dependent inflammatory function in platelets is lost in patients with sepsis resulting from community-acquired pneumonia. Further studies will need to be undertaken to determine whether this is due to localized recruitment to the lungs of a platelet responsive population or loss of function as a result of dysregulation of the immune response.

## INTRODUCTION

Platelets are critical to host defense through a multitude of direct and indirect actions (1–3), and these actions have been demonstrated in models of lung infection leading to pneumonia (4–6). However, the role of platelets in the innate immune response, hyperinflammation, and thrombosis that is associated with the pathophysiology of pneumonia, leading to acute lung injury and sepsis, is complex, and sometimes contradictory (7–10). There is therefore a need to better understand the role of platelets in these pathological sequalae in chronology, space, and amplitude (i.e., severity).

Thrombocytopenia is part of the Sequential Organ Failure Assessment score and provides prognostic information in sepsis patients. Thrombocytopenia is also associated with higher cytokine levels and endothelial cell activation, independent of disease severity (11). Animal models of pneumonia-induced sepsis recapitulate this trend, with experimental thrombocytopenia shown to increase bacterial growth, dissemination, and reduced survival (4,6). Furthermore, coagulopathies, often in the form of localized tissue thrombi (thromboinflammation), and also disseminated intravascular coagulation (DIC), occur in approximately 80% of sepsis patients (12). Thus, peripheral thrombocytopenia can be an indicator of increased platelet consumption due to DIC, but also platelet recruitment to the lung in response to inflammatory cues, independent of platelet aggregation (3,13,14), and in some instances lead to the extravascular accumulation of platelets (4,13–15), as platelets have motile (chemotactic) properties in response to pathogen-derived chemoattractants and some endogenous chemokines (14,16,17).

The role of P2Y receptors expressed on platelets leading to platelet aggregation is well established (18). However, we have reported an additional requirement for platelet P2Y_1_ receptor activation for platelet-dependent pulmonary leukocyte recruitment in the absence of changes in hemostasis (19–21), and in the case of P2Y_1_, requiring distinct signaling pathways of platelet activation (RhoA, Rac-1) leading to functional motility (chemotaxis) compared with the canonical phospholipase C pathway implicated in aggregation (20,22,23).

It has previously been suggested that platelet functions during hemostasis (e.g., aggregation) are necessarily distinct from platelet functions that occur during inflammation (e.g., chemotaxis) (24). However, these platelet functions have not been separately measured in clinical situations such as sepsis where both inflammation as a result of host defense to external pathogens and thrombotic complications occur.

Thus, platelet functions pertinent to hemostasis and inflammation were investigated in a patient population with sepsis secondary to community-acquired pneumonia (CAP) and compared with platelets isolated from patients with mild sterile inflammation resulting from elective cardiac surgery recruited to the IMMunE dysfunction and Recovery from SEpsis-related critical illness in adults (IMMERSE) observational cohort study (25). Potential changes in P2Y receptor-mediated platelet function after either mild or dysregulated inflammation could therefore be assessed. We hypothesize that platelet functions relating to hemostasis and inflammation that are P2Y_1_ mediated will be heightened during sepsis, given the increased platelet activation that is observed in such patients with respiratory infections.

## MATERIALS AND METHODS

### Materials

Acid Citrate Dextrose (ACD)-A Vacuette tubes (455055) were purchased from Greiner Bio-One (Stonehouse, United Kingdom). The purinergic receptor agonist, ADP (01905), the chemotactic peptide *N*-formylmethionyl-leucyl-phenylalanine (fMLP) (F3506) and prostaglandin E_1_ (PGE_1_) were purchased from Sigma Aldrich (Poole, United Kingdom). The HTS Transwell 96-well plates (3-μm pore size) (10077792) and RPMI 1640 cell media with l-glutamine (12004997) were purchased from Fisher Scientific (Loughborough, United Kingdom). The P2Y_1_ antagonist, MRS2500 (2,159/1), and the P2Y_12_ antagonist, AR-C66096 (3,321/1), were purchased from Bio-Techne (Minnieapolis, MN). Stromatol (321200S) was purchased from Mascia Brunelli (Milan, Italy). Tuerk’s Solution (93770) was purchased from Merck (Darmstadt, Germany).

### IMMERSE Observational Clinical Trial study design

Samples were obtained through the IMMERSE Observational Clinical Trial (IRAS project ID: 257753) (25). The study had the following three cohorts: healthy volunteers, an elective cardiac surgical cohort, and a sepsis cohort. Healthy volunteers were sampled at a single time point to act as a healthy control (HC) population. The cardiac cohort was made up of patients undergoing elective cardiac surgery for coronary artery bypass, limited to one surgical team to ensure that the anticoagulation protocol between patients was similar. Patients were of similar age and sex to sepsis patients and were sampled immediately before and 24 h after surgery, acting as a baseline and a sterile inflammatory response, respectively. The sepsis cohort comprised patients admitted to an intensive care unit (ICU) or a high dependency unit (HDU) as a consequence of a CAP, diagnosed as per the United Kingdom National Institute for Health and Care Excellence clinical practice guidance (https://www.nice.org.uk/guidance/cg191). Please note the eligibility criteria stipulated Sepsis-3 criteria for clinical diagnosis of sepsis, consistent with usual clinical practice (26). For this project, only patients with sepsis secondary to CAP were included, with samples taken at ICU/HDU admission, day 3, day 5, and when stepped down in their critical care (i.e., from ICU to HDU or the ward, or from HDU to the ward).

### Recruitment and sampling

All patients were recruited at St Thomas’ Hospital, London, part of the Guy’s and St Thomas’ NHS Trust. Cardiac cohort patients were approached and recruited by nurses from the St Thomas’ cardiology research team, and sepsis patients by the ICU research team. Samples were then taken through arterial lines using ACD-A Vacuette tubes. Eight milliliters of blood was collected within 24 h of the specified time point, stored at room temperature (18°C–23°C) and processing initiated within 2 h.

### Patient summary, healthy cohort

Healthy volunteers were recruited into the IMMERSE study to act as negative controls (n = 7, 2 male, 5 female) with an average age of 54 years (37–62 years).

### Patient summary: cardiac cohort

Patients undergoing elective cardiac surgery were also recruited into the IMMERSE study. Eight milliliters of blood was taken immediately before surgery to act as a baseline (presurgery, cardiac cohort [CC] T0) and then 24 h after surgery, when an inflammatory response resulting from the surgery was expected (CC T1). Within this cohort, 20 patients were recruited, with a median age of 70.0 years (64.0–76.5 years) and comprising 85% males and 15% females. These patients were given an Acute Physiology and Chronic Health Evaluation II score of 11.5 (10.0–13.8), remained in ICU for 3 days (2–4 days), hospital for 10 days (8.3–15.8 days), and had a 100% survival rate (Table 1).

**TABLE 1:** IMMERSE Observational Clinical Trial: cardiac cohort patient characteristics

Patient ID	Age, y	Sex	APACHE II (total)	ICU LOS, d	Hospital LOS, d	Hospital Survival
CC 01	75	M	13	6	13	Alive
CC 02	69	M	9	3	9	Alive
CC 03	82	M	10	2	9	Alive
CC 04	71	M	10	2	9	Alive
CC 05	64	M	7	2	8	Alive
CC 11	65	M	12	3	16	Alive
CC 12	43	M	11	3	8	Alive
CC 15	74	F	12	2	15	Alive
CC 16	77	F	14	5	23	Alive
CC 17	73	M	15	3	21	Alive
CC 18	64	M	10	4	39	Alive
CC 20	77	M	11	4	10	Alive
CC 21	66	M	21	3	8	Alive
CC 25	52	M	2	4	7	Alive
CC 26	77	M	19	6	10	Alive
CC 27	57	M	11	2	10	Alive
CC 28	79	M	15	2	8	Alive
CC 29	67	M	13	3	14	Alive
CC 30	72	F	12	3	18	Alive
CC 31	36	M	5	3	10	Alive

APACHE II, acute physiology and chronic health evaluation II; CC, cardiac cohort; F, female; ICU, intensive care unit; LOS, length of stay; M, male.

### Patient summary: sepsis cohort

The final group recruited to the IMMERSE trial was the sepsis cohort. Eight milliliters of blood was sampled within 24 h of ICU/HDU admission (sepsis admission, sepsis cohort [SC] T0), on day 3 (SC T1), day 5 (SC T2), and critical care step-down (SC T3). Patients in this cohort (n = 10, 6 male, 4 female) had a median age of 58.0 years (40.3–75.5 years). Although all of these patients were diagnosed with sepsis caused by CAP, a positive microbiology result could not be identified in 40% of the patients, which mirrors a recognized absence/inability to clinically confirm a proportion of microbiology results (27,28). In comparison, infection by gram-negative bacteria was found in 30% of patients, infection by gram-positive bacteria in 50% of patients, and fungal infection in 30% of patients (Table 2).

**TABLE 2:** IMMERSE Observational Clinical Trial: sepsis cohort patient characteristics

Patient ID	Ages, y	Sex	APACHE II (total)	Microbiology		ICU LOS, d	ICU survival	Hospital LOS, d	Hospital Survival
				Sample	Positive for				
SC 02	83	F	16	Drain Fluid	*Pseudomonas aeruginosa* (scant)	13	Alive	57	Alive
SC 03	19	M	9	—	—	3	Alive	13	Alive
SC 04	38	M	11	Urine	*Legionella* antigen	7	Alive	13	Alive
				BAL	*Legionella pneumophila* (scant)				
SC 05	73	M	16	—	—	4	Alive	5	Alive
SC 06	57	F	22	Urine	Pneumococcal antigen	18	Alive	25	Alive
				Blood	Streptococcus pneumoniae				
SC 07	86	M	30	—	—	32	Alive	102	Deceased
SC 11	59	M	15	—	—	4	Alive	13	Alive
SC 86	41	F	9	Blood	*Streptococcus pneumoniae* (scant)	29	Alive	40	Alive
				Blood	*Candida albicans*				
				BAL	*Candida albicans*				
SC 88	55	F	18	Sputum	*Streptococcus pneumoniae*	5	Alive	9	Alive
				Sputum	*Staphylococcus aureus*				
SC 89	63	M	22	BAL	*Candida paralilosis* (scant)	40	Deceased	40	Deceased

Gram-positive bacteria shown in purple, and gram-negative bacteria shown in pink.

APACHE II, Acute Physiology and Chronic Health Evaluation II; BAL, bronchoalveolar lavage; CC, cardiac cohort, F, female; ICU, intensive care unit; LOS, length of stay; M, male.

Patients were given an average Acute Physiology and Chronic Health Evaluation II score of 16.0 (10.5–22.0) and had a 90% survival rate. The patient who succumbed sadly passed away on day 40 of their stay in ICU. Of the surviving patients, a median ICU stay of 7.0 days (4.0–23.5 days) was observed. After critical care step-down from ICU, an additional patient unfortunately passed away after 102 days in hospital giving an overall survival rate of 80%. Of the surviving patients, a total hospital stay of 13.0 days (6.8–36.3 days) was observed (Table 2).

### Whole blood leukocyte and platelet counts

Circulating leukocytes were quantified in whole citrated blood by staining with Tuerk’s Solution (1/100). Total leukocyte concentrations were then quantified by loading 10 μL of stained blood onto an Improved Neubauer hemocytometer and manually counting leukocytes using a Leica DM 2000 LED microscope (Leica Microsystems, Wetzlar Germany) with an ×40 objective lens. In addition, blood smears were used to differentiate between neutrophils and mononuclear cells (MNCs). Five microliters of whole blood was loaded onto a clean glass slide and a separate slide at a 45-degree angle used to pull the blood across the first slide, to create a thin film across the surface of the slide. Blood smears were then stained with Shandon Kwik-Diff staining kit and cover slipped using DPX mounting media (Thermo Fisher Scientific, Waltham MA, USA). Neutrophils (PMNCs) *versus* MNCs were then quantified as a percentage and concentrations calculated using the total leukocyte concentration found earlier. Platelets were also quantified with whole blood stained with Stromatol (1/200) and platelets were manually counted using an Improved Neubauer hemocytometer and a Leica DM 2000 LED microscope fitted with an 40× objective.

### Platelet isolation

Platelets were isolated from peripheral venous blood as previously described (Amison *et al.*, 2018) (29), with minor amendments. Whole blood was centrifuged at 133*g* for 20 min at room temperature. An aliquot of the upper platelet-rich plasma (PRP) layer was then used for aggregation studies (see hereinafter). To the remaining PRP, 2.5-μM PGE_1_ was added before centrifuging at 800*g* for 10 min at room temperature. Platelet-poor plasma (PPP) was removed and used for aggregation studies (see hereinafter). The platelet pellet was resuspended in RPMI 1640 cell media, again adding 2.5 μM PGE_1_ and centrifuging at 800*g* for 10 min at room temperature. Platelets were then adjusted to a final concentration of 5 × 10^7^ platelets/mL in RPMI 1640 for chemotaxis studies (see hereinafter).

### *In vitro* platelet aggregation

Effects of ADP on platelet aggregation were quantified by light transmission aggregometry of stimulated PRP at 595 nm at 37°C using a SpectraMax 340PC shaking plate reader (Molecular Devices, San Jose CA, USA) as previously described (29). Briefly, PRP was stimulated with vehicle (PBS) or individual agonists and immediately loaded onto the plate reader. Vehicle stimulated PPP was used as a control. Measurements were taken at 15-s intervals for 16 min. In some studies, PRP was preincubated with vehicle (PBS) or increasing concentrations of the P2Y_1_-specific antagonist, MRS2550, or the P2Y_12_-specific antagonist, AR-C66096, for 10 min at room temperature before stimulation with ADP.

To evaluate the effect of platelet concentration on *in vitro* platelet aggregation, platelets were quantified in PRP taken from healthy cohort (HC) and SC samples. The effect of platelet concentration on ADP-induced *in vitro* aggregation was assessed by diluting PRP from healthy volunteers with PPP from the same donor (100:0, 75:25, 50:50, 25:75, 12.5:87.5, 5:95). Diluted PRP was stimulated with either vehicle (PBS) or 100-μM ADP and immediately loaded onto a SpectraMax 340PC shaking platelet reader (Molecular Devices). Aggregation was then measured by light transmission aggregometry, as described previously.

### *In vitro* platelet chemotaxis

Inflammatory platelet function downstream of purinergic receptor activation by ADP was also investigated through *in vitro* platelet chemotaxis as previously described, but with minor amendments (29). Washed platelets (5 × 10^7^/mL) were treated with 2-mM CaCl_2_ before stimulation with vehicle (PBS) or ADP for 5 min at room temperature. Platelets were then added to the top insert of the transwell plate, with the chemoattractant in the bottom well (0/30 nM fMLP in RPMI 1640 cell media). After 90-min incubation at 37°C, media from the bottom chamber was stained with Stromatol (1:0.5) and platelets were quantified using an improved Neubauer hemocytometer and a Leica DM 2000 LED microscope with an 40× objective lens.

### Statistical analysis

Data were tested for normality using Graphpad Prism (version 9.4.1, Graphpad Software, Boston MA, USA). Overall, data sets adhered to a normal distribution, and therefore data were presented as means with SEM. Parametric statistics were used to deduce significant differences between groups. Circulating neutrophil, MNC, and platelet numbers were analyzed by one-way ANOVA with Tukey’s multiple comparisons tests and platelets by one-way ANOVA with Dunnett’s multiple comparisons.

Platelet aggregation data were expressed with second order smoothing (4 neighbors) over the whole 16 min. Aggregation at 5 min was analyzed by ordinary two-way ANOVA with Tukey’s multiple comparisons. The correlation between platelet concentration and *in vitro* aggregation was analyzed by Pearson’s correlation coefficient. Platelet chemotaxis data was provided as a chemotactic index (CI) above basal and also analyzed by two-way ANOVA with Tukey’s multiple comparisons tests. Significant differences accepted when *P* < 0.05.

## RESULTS

### Raised peripheral circulating neutrophil counts in response to elective surgery and sepsis secondary to CAP

**Fig. 1:**
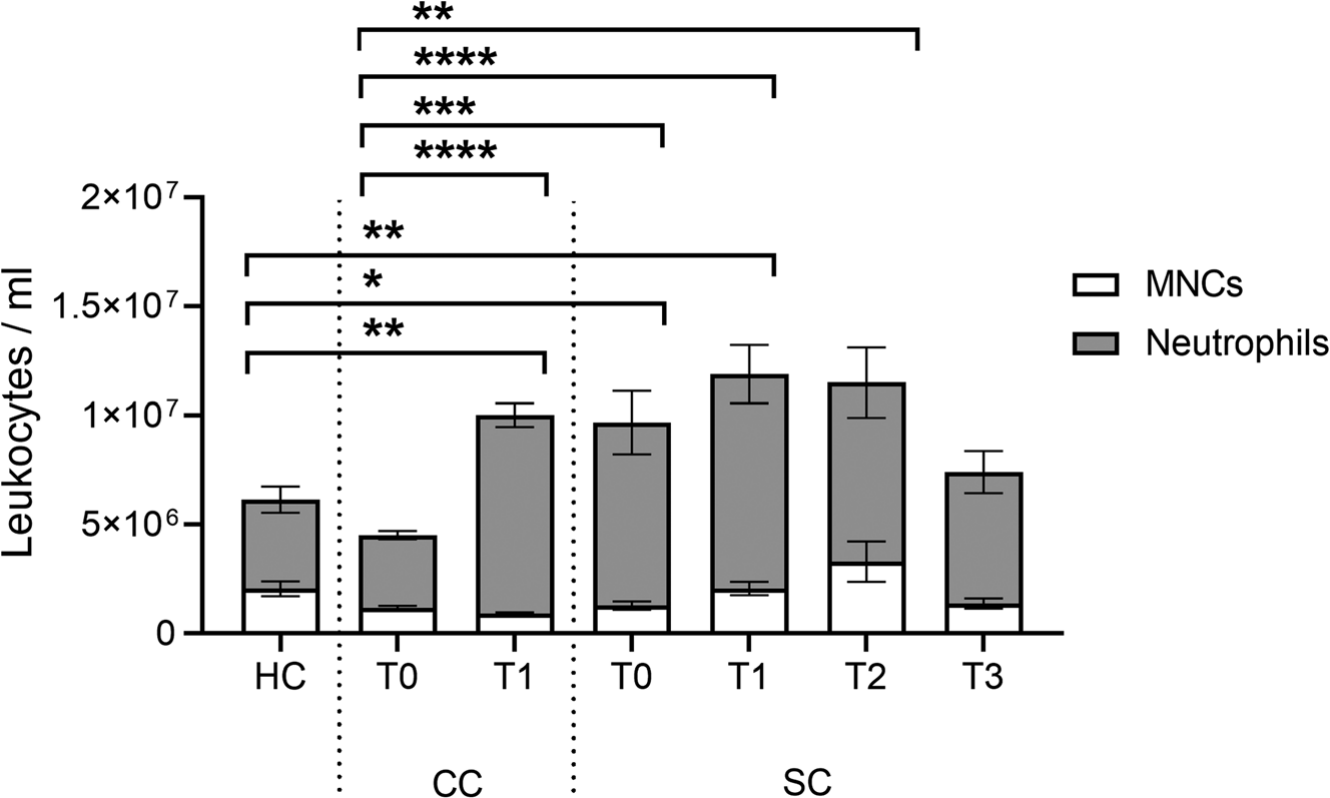
**IMMERSE Observational Clinical Trial: whole leukocyte cell counts.** Blood samples were collected from healthy donors (HC), patients undergoing elective cardiac surgery, before (CC T0) and 24 h after surgery (CC T1), and from patients admitted to critical care with sepsis secondary to CAP, sampling at admission (SC T0), day 3 (SC T1), day 5 (SC T2), and discharge (SC T3). Leukocytes were quantified in whole blood through manual counts of Tuerk’s stained blood, along with Kwik-Diff stained blood smears for differential counts of MNCs and neutrophils. Data: Means ± SEM. n = 7 (HC, SC T1), 20 (CC T0, CC T1), 10 (SC T0), 6 (SC T2), and 8 (SC T3). One-way ANOVA with Tukey’s multiple comparisons. *P < 0.05, **P < 0.01, ***P < 0.001, ****P < 0.0001 represents statistical differences between neutrophil (not MNC) counts. CAP, community-acquired pneumonia; CC, cardiac cohort; HC, healthy control; MNC, mononuclear cells; SC, sepsis cohort.

To evaluate the inflammatory response as a consequence of surgery-induced mild inflammation compared with sepsis-induced dysregulated inflammation, circulating leukocyte numbers were quantified (Fig. 1). Compared with HCs (HC: 4.1 ± 0.6 × 10^6^ cells/mL), circulating neutrophils were significantly increased in postcardiac surgery patients (CC T1: 9.1 ± 0.5 × 10^6^ cells/mL, *P* < 0.01), sepsis admission (SC T0: 8.4 ± 1.5 × 10^6^ cells/mL, *P* < 0.05), and sepsis day 3 (SC T1: 9.8 ± 1.3 × 10^6^ cells/mL, *P* < 0.01). In addition, neutrophils were significantly increased in CC T1 (*P* < 0.0001), SC T0 (*P* < 0.001), SC T1 (*P* < 0.0001), and SC T2 (*P* < 0.01) compared with presurgery controls (CC T0: 3.3 ± 0.2 × 10^6^ cells/mL). Although the cardiac cohort patients are not strictly “healthy,” here they serve as an additional age- and sex-matched negative control. Finally, the increased neutrophils observed in sepsis patients decreased by critical care discharge, with SC T3 circulating neutrophil concentrations being less than that observed at SC T1, where the neutrophil numbers were at their highest (SC T3: 6.0 ± 1.0 × 10^6^ cells/mL) (Fig. 1).

In contrast, no significant differences were observed in mononuclear cell numbers across intervention or time (HC: 2.1 ± 0.3 × 10^6^ cells/mL; CC T0: 1.1 ± 0.1 × 10^6^ cells/mL; CC T1: 0.9 ± 0.1 × 10^6^ cells/mL; SC T0: 1.3 ± 0.2 × 10^6^ cells/mL; SC T1: 2.1 ± 0.3 × 10^6^ cells/mL; SC T2: 3.3 ± 0.9 × 10^6^ cells/mL; SC T3: 1.4 ± 0.2 × 10^6^ cells/mL) (Fig. 1).

As expected, both the cardiac cohort (after elective surgery) and the sepsis cohort presented with a raised inflammatory response, recognized by neutrophilia.

### Evaluation of changes to circulating platelet counts during the inflammatory response

**Fig. 2:**
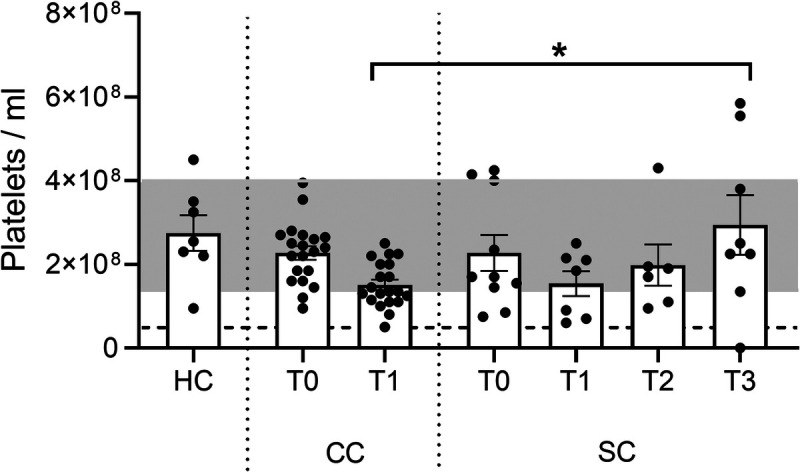
**IMMERSE Observational Clinical Trial: whole blood platelet counts.** Blood samples were collected from healthy donors (HC), patients undergoing elective cardiac surgery, before (CC T0) and 24 h after surgery (CC T1), and from patients admitted to critical care with sepsis secondary to CAP, sampling at admission (SC T0), day 3 (SC T1), day 5 (SC T2), and discharge (SC T3). Whole blood was stained using stromatol to quantify circulating platelet concentrations. Black dotted line represents clinical thrombocytopenia (<0.5 × 10^8^ platelets/mL); shaded gray area represents normal range of platelets in a healthy person (1–4 × 10^8^ platelets/mL). Data: Means ± SEM. n = 7 (HC, SC T1), 20 (CC T0, CC T1), 10 (SC T0), 6 (SC T2), and 8 (SC T3). One-way ANOVA with Tukey’s multiple comparisons. *P < 0.05. CAP, community-acquired pneumonia; CC, cardiac cohort; HC, healthy control; SC, sepsis cohort.

In addition to leukocytes, circulating platelet numbers were also investigated. Compared with HCs (HC: 2.8 ± 0.4 × 10^8^ platelets/mL) and presurgery cardiac patients (CC T0: 2.3 ± 0.2 × 10^8^ platelets/mL), platelets were nonsignificantly reduced after cardiac surgery (CC T1: 1.5 ± 0.1 × 10^8^ platelets/mL) and in sepsis patients (SC T0: 2.3 ± 0.4 × 10^8^ platelets/mL). Interestingly, although platelet numbers were reduced during sepsis, this difference was not significant, and concentrations recovered throughout ICU stay, recovering by discharge (SC T1: 1.5 ± 0.3 × 10^8^ platelets/mL; SC T2: 2.0 ± 0.5 × 10^8^ platelets/mL; SC T3: 2.9 ± 0.7 × 10^8^ platelets/mL). Indeed, platelet concentrations at SC T3 were significantly higher than that observed at SC T1 (*P* < 0.05) and were comparable with HC (Fig. 2).

Thus, although the group sizes in this study were limited, with implications for statistical analysis, elective cardiac surgery and sepsis resulted in increased circulating leukocyte concentrations—namely, they displayed an inflammatory response. Simultaneously, a corresponding reduction in platelet concentration was observed, suggestive of *in vivo* platelet recruitment and/or consumption, or decreased production.

### Platelets retain capacity for hemostatic function (*ex vivo* aggregation), in both postsurgery and sepsis patients

**Fig. 3:**
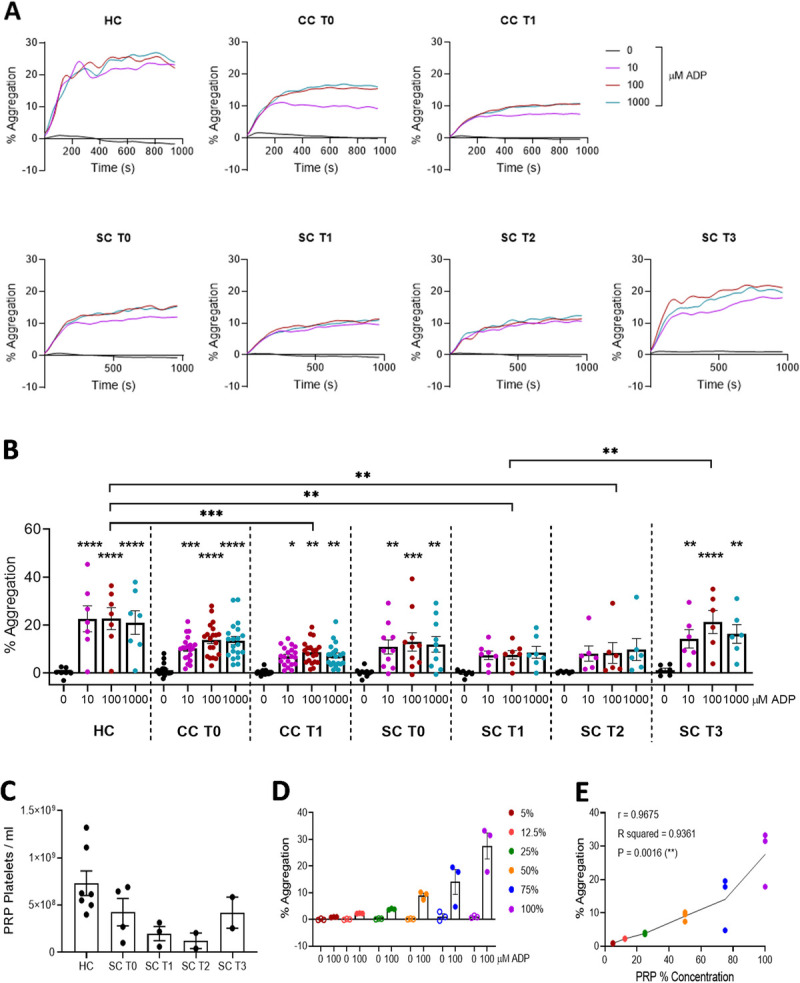
**IMMERSE Observational Clinical Trial: *ex vivo* ADP-induced platelet hemostatic function.** Platelet-rich plasma was isolated from healthy donors (HC), patients undergoing elective cardiac surgery, before (CC T0) and 24 h after surgery (CC T1), and from patients admitted to critical care with sepsis secondary to CAP, sampling at admission (SC T0), day 3 (SC T1), day 5 (SC T2), and discharge (SC T3). Platelet-rich plasma was stimulated with increasing concentrations of the P2Y_1_/P2Y_12_ receptor agonist, ADP, and platelet aggregation measured by light transmission aggregometry. A, Aggregatory traces over 16 min for each group. B, Aggregation at 5 min. C, PRP platelet concentration in different clinical groups. D, ADP-induced aggregation in PRP from healthy donors after diluting with PPP from the same donor. E, Correlation between platelet concentration in PRP and % aggregation. Pearson’s correlation coefficient. A and B, Data: Means ± SEM. n = 7 (HC, SC T1), 20 (CC T0, CC T1), 10 (SC T0), 6 (SC T2), and 8 (SC T3) per group. Two-way ANOVA with Tukey’s multiple comparisons. *P < 0.05, **P < 0.01, ***P < 0.001, ****P < 0.0001 *versus* corresponding negative control, unless stated otherwise. To ease understanding, only significant differences between 100 μM are shown. C–E, Healthy control, SC day 0 (T0), 3 (T1), 5 (T2), and discharge (T3). Data: Mean ± SEM. n = 7 (HC), 4 (SC T0), 3 (SC T1), and 4 (SC T2, SC T3). Data: Mean ± SEM. n = 3 per group. **P < 0.01. CAP, community-acquired pneumonia; CC, cardiac cohort; HC, healthy control; SC, sepsis cohort.

To investigate potential changes in hemostatic platelet function during/after an *in vivo* inflammatory insult, ADP-induced platelet aggregation was investigated *ex vivo.* Platelet-rich plasma was isolated from clinical samples and stimulated with increasing concentrations of ADP. Aggregation was then measured by light transmission aggregometry (Fig. 3A). In HCs, presurgery patients (CC T0), and samples taken from sepsis patients in the day of admission (SC T0), significant platelet aggregation occurred in response to 10, 100, and 1,000 μM ADP (Fig. 3B). Interestingly, platelet aggregation was still demonstrable in postsurgery patients (CC T1) and in sepsis patients on day 3 and 5 of admission (SC T1 and SC T2, respectively). However, these responses were significantly reduced compared with HC (Fig. 3B). Furthermore, upon discharge, platelets from sepsis patients displayed robust aggregation toward ADP, on par with HC (Fig. 3B). Because *in vitro* platelet aggregation is measured in PRP, it was noted that postsurgery and sepsis samples could be less concentrated, because they had a greater volume of PRP (data not shown), possibly because of fluid resuscitation providing a “dilution effect” on blood cell density. Furthermore, thrombocytopenia caused by localized tissue recruitment, DIC, or altered platelet production could also alter circulating platelet concentration. Therefore, the effect of platelet concentration on this assay was further investigated. First, to identify any differences in platelet concentration in the PRP obtained from different groups, platelets were quantified through manual counting of HC and SC samples. Although no significance was observed, platelet counts seemed to be reduced in sepsis patients, but normalized by the time of discharge from critical care (Fig. 3C). Based on these observations, the correlation between platelet concentration and aggregation was investigated in healthy donors. Platelet-rich plasma was isolated from healthy donors and diluted with PPP from the same donor to give either 100% PRP, 75% PRP + 25% PPP, 50%, 25%, 12.5%, or 5% PRP. Platelet-rich plasma was then stimulated with 100 μM ADP and platelet aggregation measured by light transmission aggregometry. For this experimental setup, platelet aggregation displayed a positive correlation with platelet concentration (*R*^2^ = 0.94, *P* < 0.01) (Fig. 3, D–E). Therefore, the diminutive platelet aggregatory responses observed in SC T2 and SC T3 groups (Fig. 3B) might have occurred as a result of PRP dilution due to fluid resuscitation administered to the patients.

Thus, the ability of platelets to aggregate toward ADP was conserved in patients undergoing cardiac surgery and in sepsis patients upon admission and during their stay in ICU, yet with a reduced capacity. However, it was also observed that by the date of discharge, platelet hemostatic function had recovered.

### P2Y_1_ and P2Y_12_ activity on ADP-induced platelet aggregation is conserved in both post–cardiac surgery and sepsis patients

**Fig. 4:**
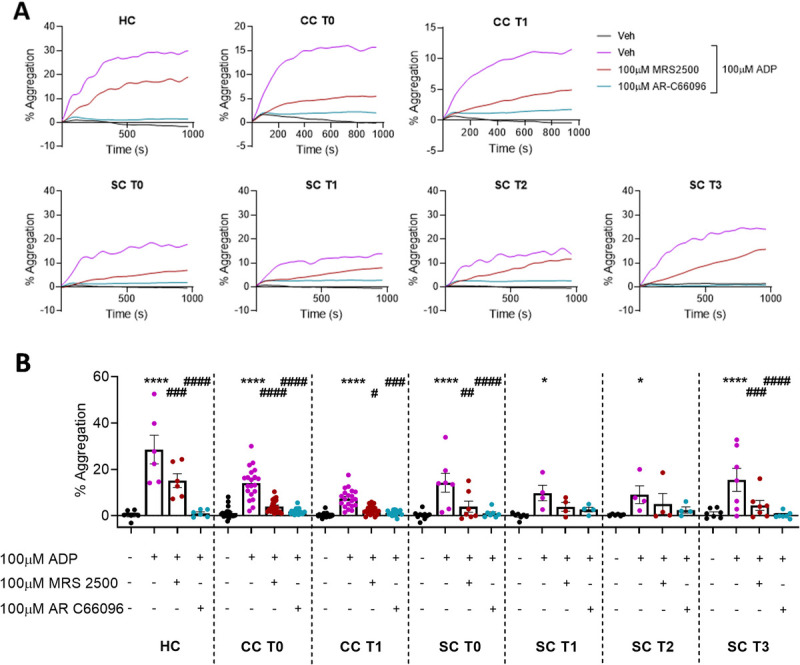
**IMMERSE Observational Clinical Trial: the mechanisms of *ex vivo* ADP-induced platelet aggregation.** As ADP can activate both the P2Y_1_ and P2Y_12_ receptors, the effect of inhibition of these receptors in ADP-induced platelet aggregation was investigated. Platelet-rich plasma was isolated from healthy donors (HC), patients undergoing elective cardiac surgery, before (CC T0) and 24 h after surgery (CC T1), and from patients admitted to critical care with sepsis secondary to CAP, sampling at admission (SC T0), day 3 (SC T1), day 5 (SC T2), and discharge (SC T3). Platelet-rich plasma was incubated with antagonists against either P2Y_1_ (MRS 2500) or P2Y_12_ (AR-C66096) before stimulating with ADP. Platelet aggregation was then measured by light transmission aggregometry. A, Aggregatory traces over 16 min for each group. B, Aggregation at 5 min. Data: Means ± SEM. C, n = 7 (HC, SC T1), 20 (CC T0, CC T1), 10 (SC T0), and 6 (SC T2, SC T3) per group. Two-way ANOVA with Tukey’s multiple comparisons. *P < 0.05, **P < 0.01, ***P < 0.001, ****P < 0.0001 *versus* corresponding negative control, unless stated otherwise. #*P* < 0.05, ##*P* < 0.01, ###*P* < 0.001, ####*P* < 0.0001 compared with corresponding positive control. CAP, community-acquired pneumonia; CC, cardiac cohort; HC, healthy control; SC, sepsis cohort.

ADP elicits platelet aggregation through both P2Y_1_ and P2Y_12_ receptor activation, and this was assessed in platelets isolated from the different patient groups. Before stimulation with 100 μM ADP, PRP was incubated with either MRS2500 or AR-C66096 to inhibit either P2Y_1_ or P2Y_12_ receptors respectively. Similar to previous data presented previously, 100 μM ADP elicited significant aggregation in platelets isolated from HC, CC T0, CC T1, SCT0, and SC T3 groups and to a lesser degree SC T1 and SC T2 groups (Fig. 4, A and B). Within the CC T1, SCT0 and SC T3 groups, incubation of platelets with either MRS2500 or AR-C66096 inhibited aggregation in a significant manner, and with a similar quality to the HC and CC T0 groups (Fig. 4B). Overall, these data show that the requirement for P2Y_1_- and P2Y_12_-dependent aggregation toward ADP to be conserved across the different patient cohorts, both qualitatively and quantitatively.

### P2Y_1_-dependent platelet chemotaxis is lost in sepsis patients

**Fig. 5:**
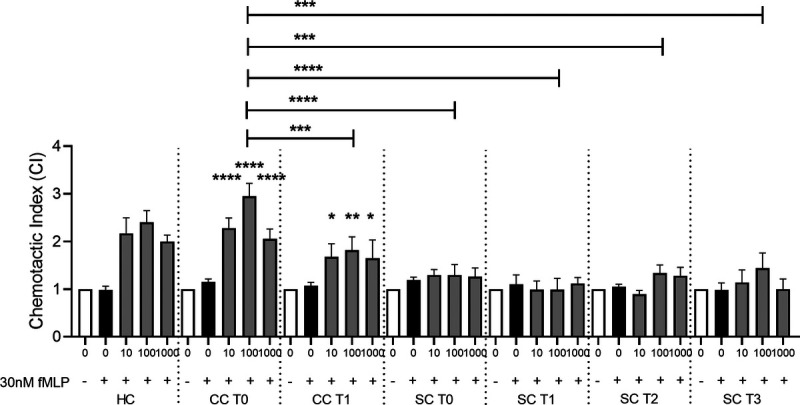
**IMMERSE Observational Clinical Trial: *ex vivo* ADP-induced platelet inflammatory function.** Platelets were isolated from healthy donors (HC), patients undergoing elective cardiac surgery, before (CC T0) and 24 h after surgery (CC T1), and from patients admitted to critical care with sepsis secondary to CAP, sampling at admission (SC T0), day 3 (SC T1), day 5 (SC T2), and discharge (SC T3). Washed platelets (5 × 10^7^/mL) were then stimulated with increasing concentrations of ADP, and platelet chemotaxis toward fMLP was measured using a transwell assay setup. Data: Means ± SEM. n = 3 (HC), 19 (CC T0), 18 (CC T1), 10 (SC T0), 6 (SC T2), 7 (SC T1), and 8 (SC T3) per group. Two-way ANOVA with Tukey’s multiple comparisons. *P < 0.05, **P < 0.01, ***P < 0.001, ****P < 0.0001 *versus* corresponding negative control, unless otherwise stated. CAP, community-acquired pneumonia; fMLP, *N*-formylmethionyl-leucyl-phenylalanine; CC, cardiac cohort; HC, healthy control; SC, sepsis cohort.

In addition to ADP-mediated hemostatic function, potential changes in *ex vivo* inflammatory platelet function (chemotaxis) were also investigated, which we have previously reported to be P2Y_1_ dependent (20,23,29). Washed platelets were isolated from clinical samples and stimulated with ADP, and chemotaxis toward fMLP was measured after 90 m (Fig. 5). In the HC, a trend of ADP-induced chemotaxis was observed, peaking at 100 nM (mean CI = 2.4 ± 0.2). Although this data set did not reach statistical significance (*P* = 0.08 compared with negative control, n = 3), it does provide a comparison of the amplitude of the signal with platelets isolated and prepared under the conditions experienced with the constraints of this clinical trial setting, with that of our previously published data (20,23) (Fig. 5). Thus, in CC T0, chemotaxis was observed after *ex vivo* stimulation by 10 nM (mean CI = 2.3 ± 0.2, *P* < 0.0001 compared with negative control), 100 nM (mean CI = 2.9 ± 0.3, *P* < 0.0001), and 1,000 nM (mean CI = 2.1 ± 0.2, *P* < 0.0001), with the highest effect size at 100 nM ADP (Fig. 5), and analogous to the HC data. Furthermore, in the CC T1 group, chemotaxis was preserved after *ex vivo* stimulation by 10 nM (mean CI = 1.7 ± 0.3, *P* < 0.05), 100 nM (mean CI = 1.8 ± 0.3, *P* < 0.01), and 1,000 nM ADP (mean CI = 1.6 ± 0.4, *P* < 0.05), with the highest effect size also seen at 100 nM ADP (Fig. 5). In contrast, no significant ADP-induced platelet chemotaxis was observed in samples from sepsis patients at any time point (Fig. 5). Comparing between groups for 100 nM ADP *ex vivo* stimulation, no differences were observed between HC and CC T0. However, chemotaxis was significantly reduced in CC T1 (*P* < 0.001), SC T0 (*P* < 0.0001), SC T1 (*P* < 0.0001), SC T2 (*P* < 0.001), and SC T3 (*P* < 0.001), compared with presurgery CC T0 controls (Fig. 5). Thus, compared with the aggregatory function of platelets, which was present upon ICU admission for pneumonia-induced sepsis patients (SC T0), and restored upon discharge (SC T3), the chemotaxis function of platelets in this patient cohort was absent and never recovered, despite this activity also being P2Y_1_ dependent.

## DISCUSSION

To our knowledge, this is the first clinical observational study comparing a distinct inflammatory function to that of the hemostatic function of platelets isolated from a cohort of sepsis patients, and also from a cardiac surgery cohort as a comparator for sterile inflammation. Platelets taken from sepsis patients retained, to some capacity, their ability to aggregate in response to ADP *via* P2Y_1_ and P2Y_12_ receptor stimulation. While it has been reported elsewhere that there is a reduced platelet function in sepsis patients (30), our study highlights that there are important experimental considerations to consider. The *in vitro* assay involves stimulating PRP and measuring platelet aggregation within this medium, which is affected by the platelet concentration within the PRP and the percentage of aggregation measured in this assay. Within the PRP of patients who had undergone cardiac surgery and, more strikingly, sepsis patients, the volume of plasma was greatly increased. This effect was likely a result of the fluid resuscitation strategies performed during surgery and in sepsis. Thus, we consider the demonstrable platelet aggregation within the sepsis cohort, while diminished compared with HC, and CS T0 groups, provided evidence that this aggregatory function was retained.

In contrast, the P2Y_1_-dependent function of platelet chemotaxis was absent in these patients (an assay which is not affected by platelet density in PRP). We have previously reported that the stimulation of platelets with ADP *via* P2Y_1_ receptors to induce either chemotaxis (toward a chemoattractant) or aggregation requires qualitatively different signaling pathways (aggregation is phospholipase C dependent, whereas chemotaxis is RhoA and Rac-1 dependent) and is a demonstration of the dichotomy of platelet function (21,23,24). Animal models of pulmonary inflammation further indicate that the platelet P2Y_1_-RhoA/Rac-1 pathway is important for platelet and leukocyte recruitment in response to both allergens and pathogen-derived mediators (20,22). The reasons for the absence of this *ex vivo* chemotactic response are unclear, although increased platelet activation is found in many inflammatory conditions, including sepsis (11). *In vivo* platelet activation could help explain the decreased *ex vivo* platelet chemotaxis we observed. For example, platelet recruitment to sites of inflammation is a phenomenon that has been described extensively (3,13–15), and thus platelets responsive to chemotactic cues might have been absent or reduced in the circulation. In support of this, it has been reported that different platelet phenotypes exist within the body, and these different population subsets respond differentially to certain stimuli (31) and possibly also in sepsis patients (32).

Alternatively, *in vivo* platelet activation may lead to an exhausted platelet phenotype, meaning the functionality of those platelets is then reduced and they are unable to undergo chemotaxis *ex vivo*. Nevertheless, this scenario does not explain why hemostatic function of platelets *in vitro* was differentially maintained in patients with sepsis where the cells would have been in an *in vivo* environment associated with DIC. Moreover, it is interesting that the activation of platelets *in vivo*, in an inflammatory setting, did not lead to an increase in the amplitude or sensitivity of platelets to aggregate to ADP *ex vivo.* It is possible that a “nucleotide” halo determines platelet function in inflammation compared with hemostasis, *via* interaction between P2Y_1_ and P2Y_12_ signaling, as has been demonstrated between P2Y_1_ and P2Y_12_ to balance platelet activation and inhibition during hemostasis (18,33). Another hypothesis could be that these sepsis patients remained significantly immunosuppressed and that this impacted on proinflammatory platelet function and might suggest why platelet motility did not recover at the time of patient discharge (SC T3). While nucleotide activation of platelets is important during both hemostasis and inflammation and was the hypothesis being tested in this study, it is recognized that platelet responses to other agonists that are increased during trauma (e.g., thrombin) should also be investigated.

A significant limitation of this study is the low number of patients recruited in the sepsis cohort. This was due to the impact of the COVID-19 epidemic during 2020 and 2021 restricting our ability to obtain samples. This limitation may account for the nonsignificant incidence of thrombocytopenia reported here, compared with previous studies where thrombocytopenia acts as a negative prognostic marker in sepsis patients, with lower circulating platelet counts correlating with disease severity and mortality (11,34). Thrombocytopenia has previously been found to occur in 20% to 50% of patients (35–37), which would correspond to between two and five patients in this study. Although no patients had a circulating platelet count of less than 5 × 10^7^ platelets/mL at admission, one patient at day 3 had a platelet count very close to clinical thrombocytopenia, at 6 × 10^7^ platelets/mL. This could suggest the patients recruited to this study had milder than average disease, although the ICU length of stay (LOS) of 7.0 days (4.0–23.5 days) was longer than observed in other studies. For example, one study found 25.3% of 304 sepsis patients admitted to ICU were already thrombocytopenic, with a further 22.3% developing thrombocytopenia during ICU stay, and these patients had an average ICU stay of 3.1 days (1.6–7.8 days) (37). Furthermore, circulating platelet counts in sepsis patients at admission did not correlate with ICU LOS in this data set, again likely due to the low n numbers recruited to the sepsis cohort. Furthermore, platelet count in the postsurgery cardiac patients showed no correlation with ICU LOS. However, ICU LOS was short in this group, with a median stay of 3 days (2–4 days).

A further limitation of this study is that the *in vitro* methods of platelet aggregation and chemotaxis do not encompass the extensive actions and participation of platelets during hemostasis and immune responses, respectively, but only mimic specific events that occur *in vivo*. Platelet activation in hemostasis controls fibrin production, fibrinogen binding as part of the aggregation phenomenon, granule secretion (for example ATP, ADP, 5-HT), prostanoid (TXA2) synthesis, and clot retraction, while activation during host defense and inflammation encompasses aggregation-independent adhesion, extravascular migration, phagocytosis, modulation of inflammasome activity, and communication with other immune cells.

Overall, these data further consolidate the idea that platelets are also inflammatory cells that may contribute to the pathophysiology of sepsis and thus could represent important potential therapeutic targets for the treatment of this condition. Unsurprisingly, current antiplatelet therapies focus on inhibiting mechanisms traditionally thought to mediate hemostatic platelet function, but this and other studies suggest distinct control of hemostastic and inflammatory functions of platelets, and novel therapies targeting these distinct functions may be more beneficial in certain scenarios to counter inappropriate activation of platelets (24,38,39).

Authors’ contributions: KLA, MF, and AJ designed and performed the research, analyzed the data, and wrote the manuscript. CPP designed research and helped write the manuscript. MS-H designed research, produced and directed the IMMERSE Observational Trial, and helped write the manuscript. SCP proposed the project, designed research, and wrote the first draft of the manuscript.

## DATA AVAILABILITY

The data that support the findings of this study are available from the corresponding author upon reasonable request. Some data may not be made available because of intellectual property rights, privacy, or ethical restrictions
